# Discussion of the classification of pediatric drug clinical trials in children's hospitals in China

**DOI:** 10.3389/fmed.2024.1409270

**Published:** 2024-07-01

**Authors:** Yong Yang, Rui Jiang, Gengliang Bai, Qingqing Liu, Yongfa Chen

**Affiliations:** ^1^School of International Pharmaceutical Business, China Pharmaceutical University, Nanjing, Jiangsu, China; ^2^School of Health Economics and Management, Nanjing University of Chinese Medicine, Nanjing, Jiangsu, China; ^3^Nanjing Children's Hospital Affiliated to Nanjing Medical University, Nanjing, Jiangsu, China

**Keywords:** pediatric drug, clinical trials, children's hospital, drug research, children's medicine, clinical research

## Abstract

**Objectives:**

This study aimed to gain insights into pediatric clinical trials conducted in children's hospitals in China and provide valuable references for the development of children's hospitals and the research and development of pediatric drugs.

**Methods:**

A comprehensive search was performed on the Chinese Clinical Trial Registry (Chi CTR) and ChinaDrugTrials.org.cn to collect information on all clinical trials involving subjects under 18 years, including those conducted in children's hospitals. The retrieval period was extended until 31 December 2022.

**Results:**

A total of 459 pediatric clinical trials were collected, comprising 299 from Chi CTR and 160 from the Drug Clinical Trial Registration and Information Publicity Platform (Information Platform). Post-marketing drug studies and phase III clinical trials accounted for the majority of research stages. These trials covered a wide range of diseases/systems, with a particular focus on respiratory system disorders, tumors, endocrine disorders, and nutritional or metabolic diseases. Chemical drugs constituted the most extensively studied category, while traditional Chinese medicine/natural drugs received comparatively less attention. Clinical trial activities were primarily geographically focused on the eastern coastal regions of China, with multicenter trials being the most predominant. Ethics committee approval was obtained for 427 studies.

**Conclusion:**

The pediatric clinical trials conducted by children's hospitals in China have shown an overall upward trend; however, there is limited research focusing on traditional Chinese medicine, along with significant regional and institutional imbalances. Furthermore, there is still room for improvement regarding ethical review processes. It is recommended that children's hospitals enhance their scientific research capabilities while optimizing resource allocation to meet medical service demands effectively. Additionally, fostering more research-focused children's hospitals will contribute to the high-quality development of children's health in China.

## 1 Introduction

In the context of “Healthy China” and fertility promotion policies, China is set to enhance its investment in children's healthcare, bolstering the sector as a pivotal element in healthcare improvement. This increase in resources is expected to catalyze advancements in scientific research at children's hospitals, thereby enhancing the level of child health protection in China. In terms of medical institutions, a children's hospital, as a specialized pediatric hospital, is the main channel through which children can obtain medical services, and it is very important to promote the all-around and high-quality development of children's hospitals. However, China faces a notable challenge with a severe shortage of pediatric medications and children's use of drugs beyond the instructions (1). Pediatric drug clinical trials are important links before children's medication are sold on the market. Developing pediatric drug clinical trials and promoting children's medication on the market can effectively address the fundamental issues underlying the shortage of children's medication (2).

The term “clinical trial” refers to a prospective study wherein subjects are administered one or more health-related interventions (such as drugs, medical devices, and behavioral interventions) to assess the impact of these interventions on human health outcomes (3). Registering clinical trials is an ethical imperative in medical research, and it is explicitly mandated by the Helsinki Declaration that “every clinical trial must be registered in a publicly accessible database prior to enrolling the first subject.” Furthermore, relevant legislation stipulates that clinical trials must undergo registration (4, 5). Currently, the primary platforms for clinical trial registration in China include the Chinese Clinical Trial Registry (Chi CTR) at www.chictr.org.cn and the Drug Clinical Trial Registration and Information Publicity Platform (Information Platform) at www.chinadrugtrials.org.cn.

Scholars in China have discovered that pediatric drug clinical trials have experienced rapid development, primarily focusing on chemical drugs and biological products. However, compared to developed countries, the number and structural proportion of projects remain relatively small (6–12). Based on research needs, children's hospitals refer specifically to specialized pediatric hospitals, excluding outpatient departments in general hospitals, maternal and child health hospitals, and women's and children's hospitals. Currently, limited research is available regarding the current status of pediatric drug clinical trials conducted within children's hospital systems or comparative studies across different clinical trial registration platforms. Therefore, this study aims to analyze the pediatric drug clinical trials carried out within China's children's hospital system by collecting information from the Chi CTR and Information Platform. The objective is to provide valuable insights for Chinese children's hospitals to enhance their participation in the research and development of pediatric drugs.

## 2 Materials and methods

### 2.1 Materials

On the platform of Chi CTR, the research type is “intervention research,” and the unit (hospital) in the implementation place searches “Children,” “Children's Hospital,” “Pediatric Hospital,” and “Children's Medical Center” until 31 December 2022. On the Information Platform, clinical participating institutions searched for “Children,” “Children's Hospital,” “Pediatric Hospital,” and “Children's Medical Center” until 31 December 2022.

### 2.2 Inclusion and exclusion criteria

Inclusion criteria: The subjects are younger than 18 years; the research content is related to the pediatric population and drugs. The research type of the Information Platform project is “intervention research.” The research implementation unit includes at least one children's hospital; the state of the experiment, the stage of the experiment, and the diseases studied are not limited.

Exclusion criteria: The subjects are older than 18 years. The research content is irrelevant to the pediatric population or drugs (such as medical devices and behavioral interventions) and non-intervention research.

### 2.3 Methods

We checked the retrieval results one by one, screened the clinical trial projects according to the above inclusion and exclusion criteria, and recorded the relevant information of the included projects, including project name, registration time, trial status, trial phase, responsible unit for research implementation, and research design. The classification of diseases/systems involved in the study refers to the 11th revision of the International Classification of Diseases (ICD-11), and research types and design methods refer to epidemiology (13). Excel 2021 software was used to input and standardize data and perform descriptive analyses.

## 3 Results

Initially, we searched the Chi CTR and Information Platform, individually checking information on each item and selecting items according to the inclusion and exclusion criteria. Finally, Chi CTR included 299 related clinical trials (out of all 305 items, six items were duplicates registered on both platforms and were excluded), and the Information Platform included 160 related clinical trials, totaling 459 items.

### 3.1 Trial registration time, quantity distribution, and status

**Figure 1:**
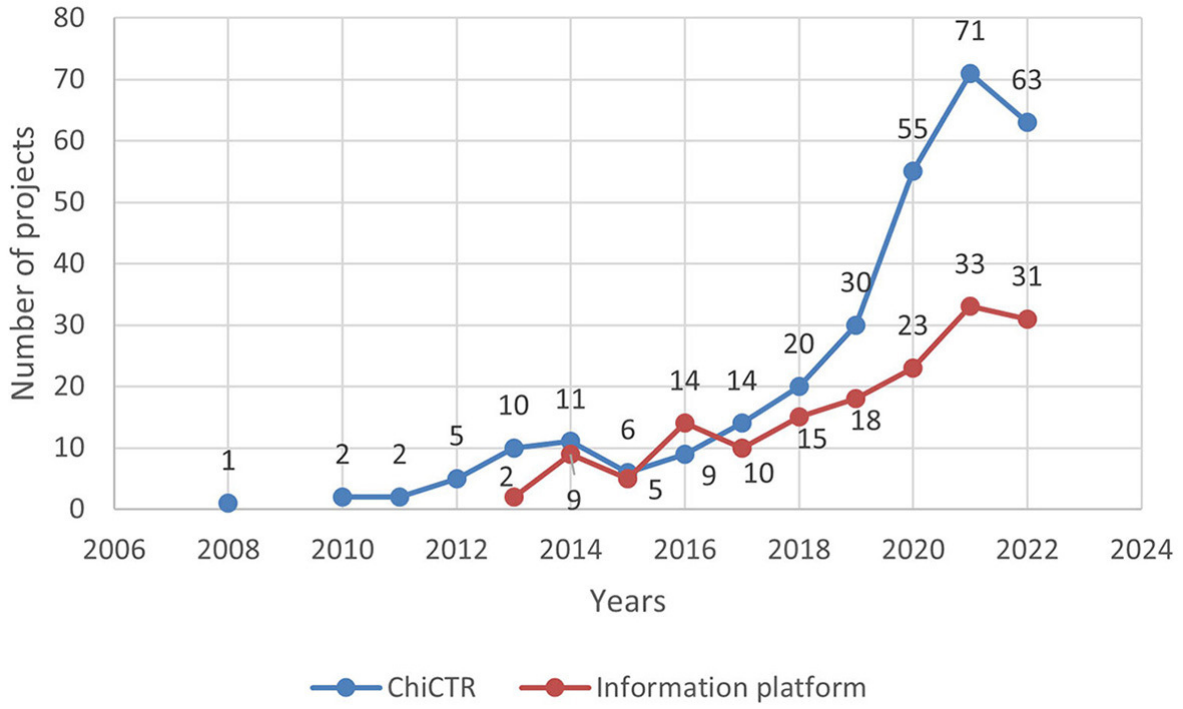
Time-number distribution of pediatric drug clinical trials in children's hospitals in China.

Chi CTR was established in 2005, and the Information Platform was officially launched in 2013. Among them, in the years 2005, 2007, and 2009, Chi CTR did not publish any related clinical trials conducted in children's hospitals, as shown in Figure 1. As of 31 December 2022, the relevant test status of each platform is shown in Table 1.

**Table 1:** Status of pediatric drug clinical trials in children's hospitals in China.

**Platform**	**Clinical trial status**	**Number**
Chi CTR	Ongoing	143 (47.83%)
	Not yet recruiting	112 (37.46%)
	Completed	44 (14.72%)
Information Platform	Not yet recruiting	29 (18.13%)
	Recruiting	62 (38.75%)
	Completed recruitment	25 (15.63%)
	Completed	38 (23.75%)
	Suspended	2 (1.25%)
	Terminated	4 (2.50%)

### 3.2 The phase of the clinical trial

**Figure 2:**
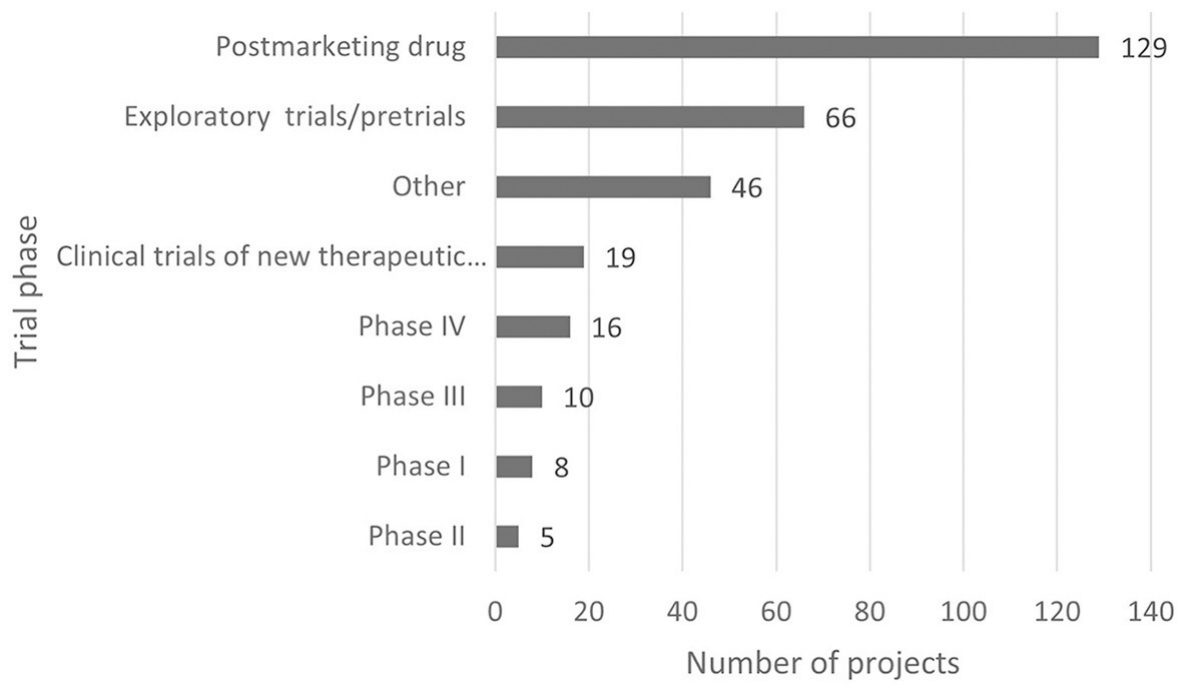
Distribution of clinical trials on Chi CTR.

**Figure 3:**
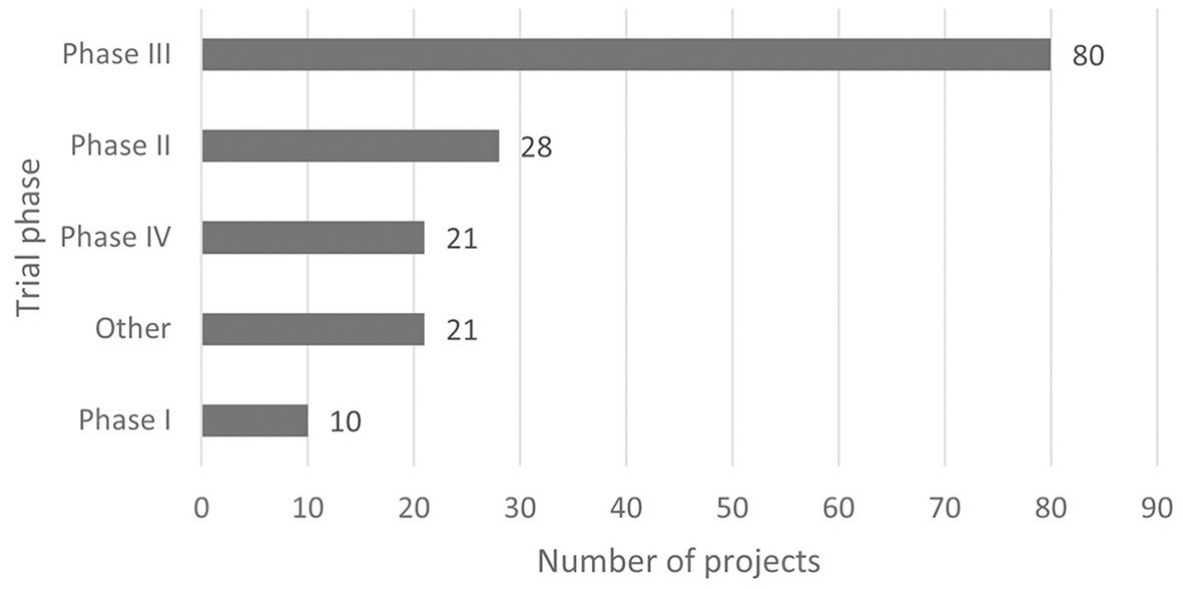
Distribution of clinical trials on the Information Platform.

According to the classification criteria of clinical trials based on the Chi CTR and Information Platform, the distribution of pediatric drug clinical trials in children's hospitals in China is shown in Figures 2, 3. Exploratory trial/pre-trial refers to the clinical verification of unlisted new drugs in the human body in a short time, with a small sample and a small dose, to investigate the reproducibility of human pharmacokinetic/pharmacodynamic parameters of preclinical data and provide a basis for further drug research and development (14). The trial of new treatment technology includes a combination of drugs and other interventions. Chi CTR's “Other” means that the experimental stage of the research project is not within the scope of the optional research stage of the platform; Information Platform's “Other” refers to all clinical trial stages except for phases I, II, III, and IV, such as post-marketing research, bioequivalence testing, and clinical verification. As can be seen from Figures 2, 3, 129 clinical trials of Chi CTR (accounting for 43.14%) are post-marketing drugs, and 80 clinical trials of Information Platform (accounting for 50%) are in Phase III.

### 3.3 Disease/system distribution in clinical trials

The clinical trials involved in this study refer to ICD-11, involving 17 diseases/systems. Another 84 clinical trials related to the anesthetic sedation research of drugs (accounting for 18.30%) are not listed in the table. The diseases/systems involved in clinical trials are respiratory system, tumor, endocrine nutrition, or metabolic diseases. Among them, the respiratory system is the most involved disease/system in Chi CTR and Information Platform research (Table 2).

**Table 2:** Distribution of diseases/systems in pediatric drug clinical trials in children's hospitals in China.

**Diseases/systems**	**Number**			**Total proportion**
	**Chi CTR**	**Information Platform**	**Total**	
Respiratory system	50	37	87	23.20
Tumor	33	12	45	12.00
Endocrine, nutritional, or metabolic diseases	7	31	38	10.13
Digestive system	27	9	36	9.60
Nervous system	15	15	30	8.00
Diseases of the blood or hematopoietic organs	13	11	24	6.40
Mental, behavioral, or neurodevelopmental disorders	11	9	20	5.33
Immune system	11	4	15	4.00
Circulatory system	9	5	14	3.73
Infectious diseases	13	—	13	3.47
Dysplasia	5	8	13	3.47
Skin diseases	7	3	10	2.67
Urogenital system	10	—	10	2.67
Musculoskeletal system	3	6	9	2.40
Visual system	7	2	9	2.40
Oral system	2	—	2	0.53

### 3.4 Distribution of clinical trial drug types

**Figure 4:**
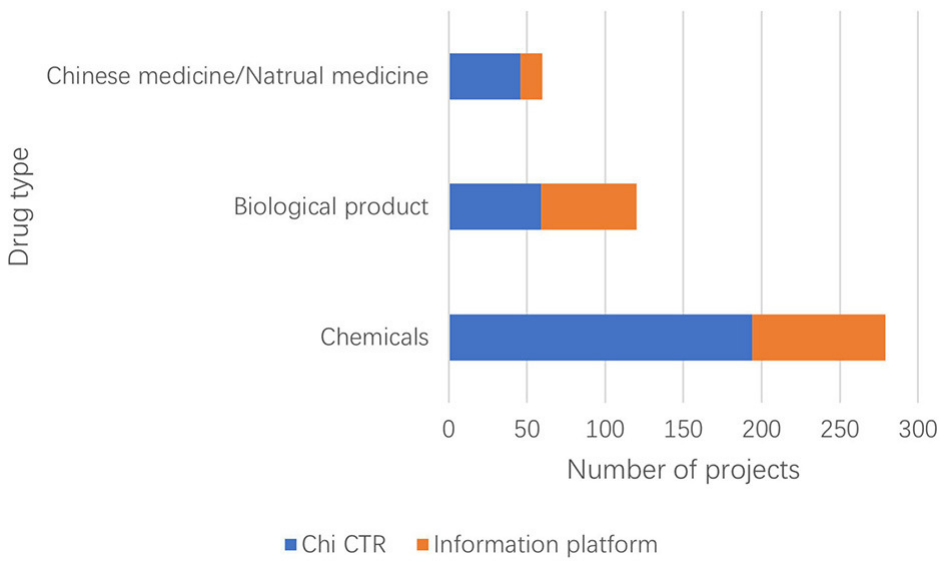
Types of drugs for pediatric drug clinical trials in children's hospitals in China.

The Information Platform requires the registration information of clinical trial items to include the types of drugs studied and divides them into three types: chemical drugs, biological products, and traditional Chinese medicine/natural drugs. The clinical trial registration information of Chi CTR does not include “drug type,” and this study confirmed the drug types of the research projects included in the platform individually (Figure 4).

### 3.5 Age-related distribution of subjects

**Figure 5:**
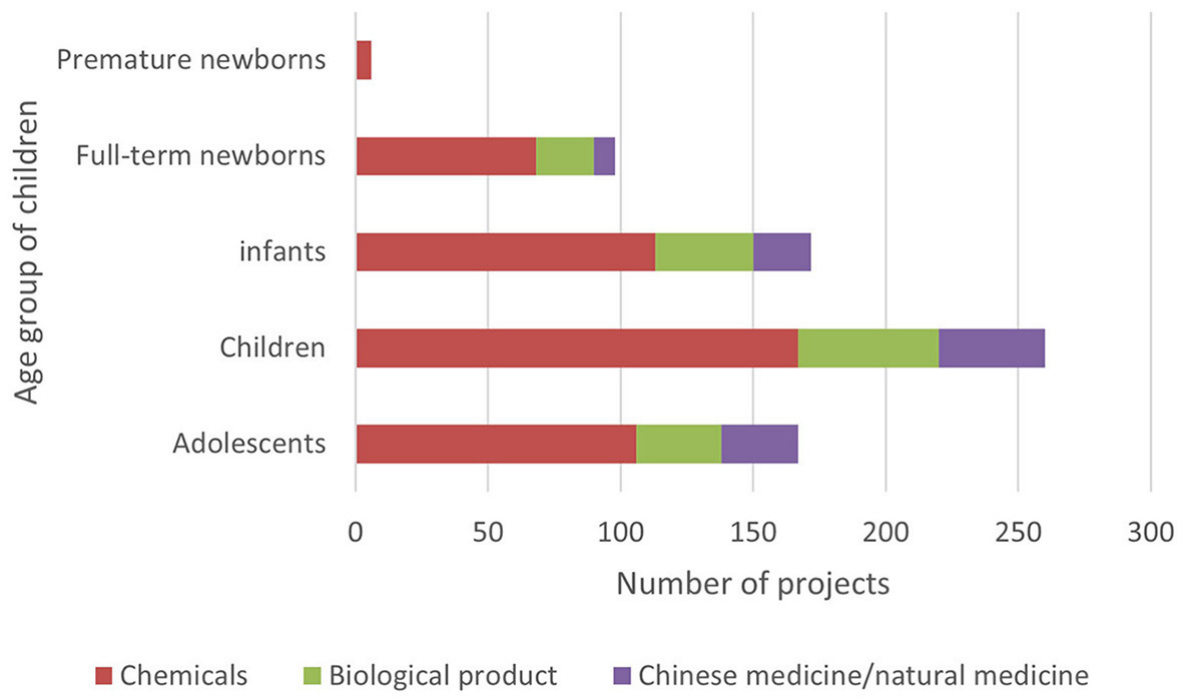
Age distribution of subjects in clinical trials on Chi CTR (drug type).

**Figure 6:**
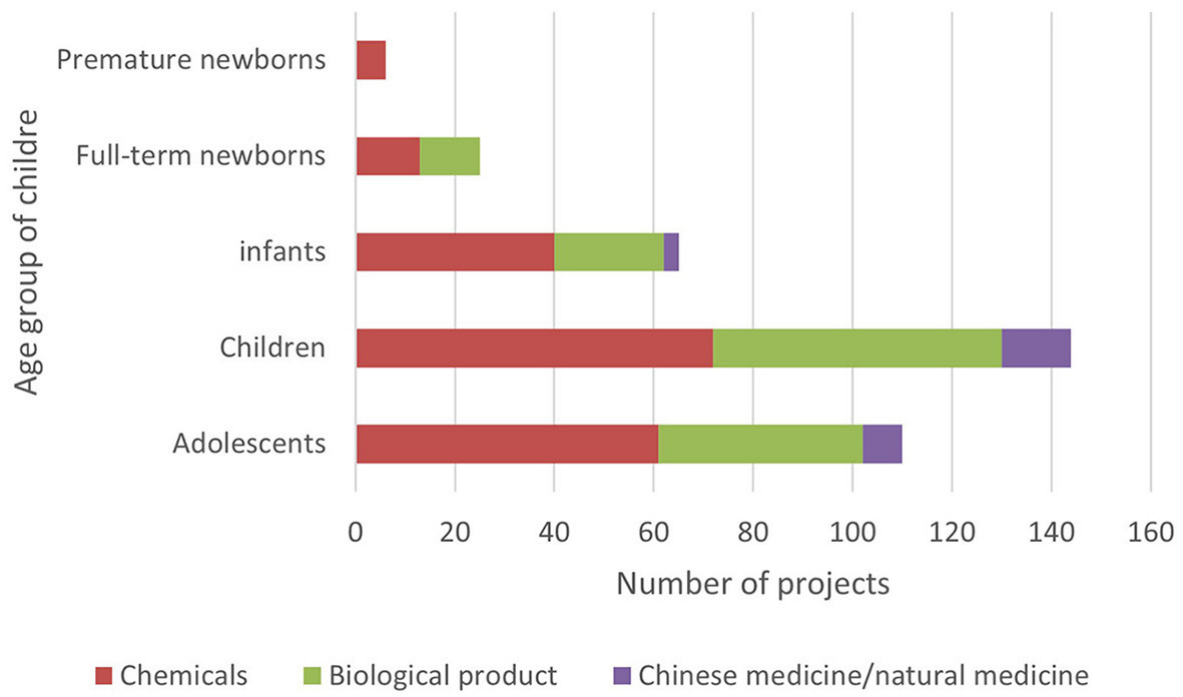
Age distribution of subjects in clinical trials on the Information Platform (drug type).

**Figure 7:**
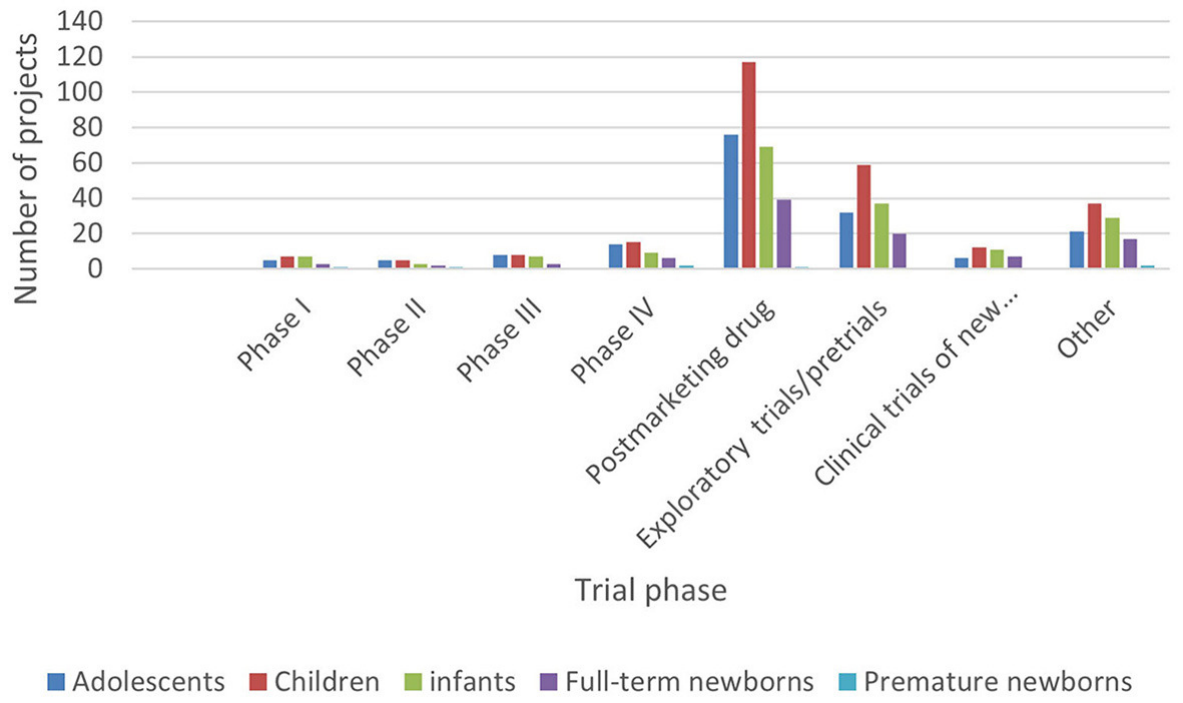
Age distribution of subjects in clinical trials on Chi CTR (clinical trial phase).

**Figure 8:**
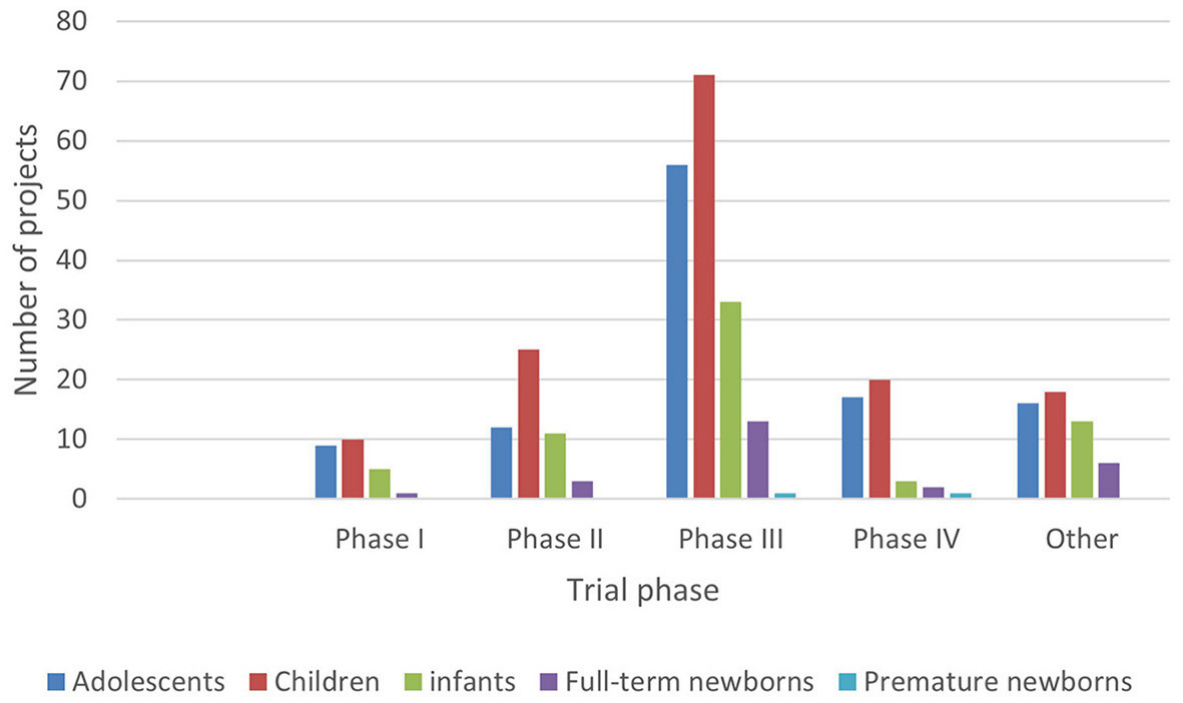
Age distribution of subjects in clinical trials on the Information Platform (clinical trial phase).

In ICH E11, pediatric patients are divided into premature newborns, full-term newborns (0–27 days), infants (28 days−23 months), children (2–11 years old), and adolescents (12–18 years old) (15). In order to reflect the pediatric drug clinical trial for pediatric patients of different ages, this study combined the age of the subjects with the types and phases of drugs in clinical trials (Figures 5–8).

### 3.6 Geographical distribution of clinical trial implementation units

According to the leader unit of a clinical trial project (the unit mainly responsible for research and implementation) and the area where the participating units are located, the regional distribution of children's hospitals is counted. There are 278 clinical trial projects led by Children's Hospital (62.61%, 278/444), 217 by Chi CTR (72.58%, 217/299), and 82 by Information Platform (51.25%, 82/160). As can be observed from Table 3, the number of pediatric drug clinical trial projects undertaken or participated in by children's hospitals in Beijing, Shanghai, Chongqing, Zhejiang, and Jiangsu ranks among the top five in China, and the number of clinical trial projects carried out by children's hospitals in the above five regions as team leaders account for 52.07% (239/459) of the total projects. Table 4 further analyzes the top five children's hospitals that carry out pediatric drug clinical trials.

**Table 3:** The top 10 regions in the number of pediatric drug clinical trials.

**Team leader unit**				**Participating unit**			
**Regions**	**Chi CTR**	**Information platform**	**Total**	**Regions**	**Chi CTR**	**Information platform**	**Total**
Beijing	47	54	101	Shanghai	47	85	132
Shanghai	33	16	49	Jiangsu	48	82	130
Chongqing	38	1	39	Beijing	36	79	115
Zhejiang	22	10	32	Zhejiang	11	57	68
Jiangsu	18	—	18	Chongqing	22	37	59
Guangdong	16	—	16	Henan	19	35	54
Shandong	9	1	10	Jiangxi	13	35	48
Hunan	10	—	10	Guangdong	22	24	46
Hebei	4	—	4	Hunan	14	27	41
Henan	3	—	3	Shanxi	1	32	33

**Table 4:** The top five units in the number of pediatric drug clinical trials.

	**Institution**	**Number**		
		**Chi CTR**	**Information platform**	**Total**
Team leader unit	Beijing Children's Hospital of Capital Medical University	39	51	90
	Children's Hospital of Chongqing Medical University	38	1	39
	Children's Hospital of Zhejiang University Medicine School	22	10	32
	Pediatric Hospital of Fudan University	15	10	25
	Shenzhen Children's Hospital	16	—	16
Participating unit	Children's Hospital of Zhejiang University Medicine School	9	57	66
	Beijing Children's Hospital of Capital Medical University	22	39	61
	Children's Hospital of Chongqing Medical University	22	37	58
	Children's Hospital of Shanghai	20	35	55
	Children's Hospital of Suzhou University	21	32	53

### 3.7 The scope of clinical trials

**Figure 9:**
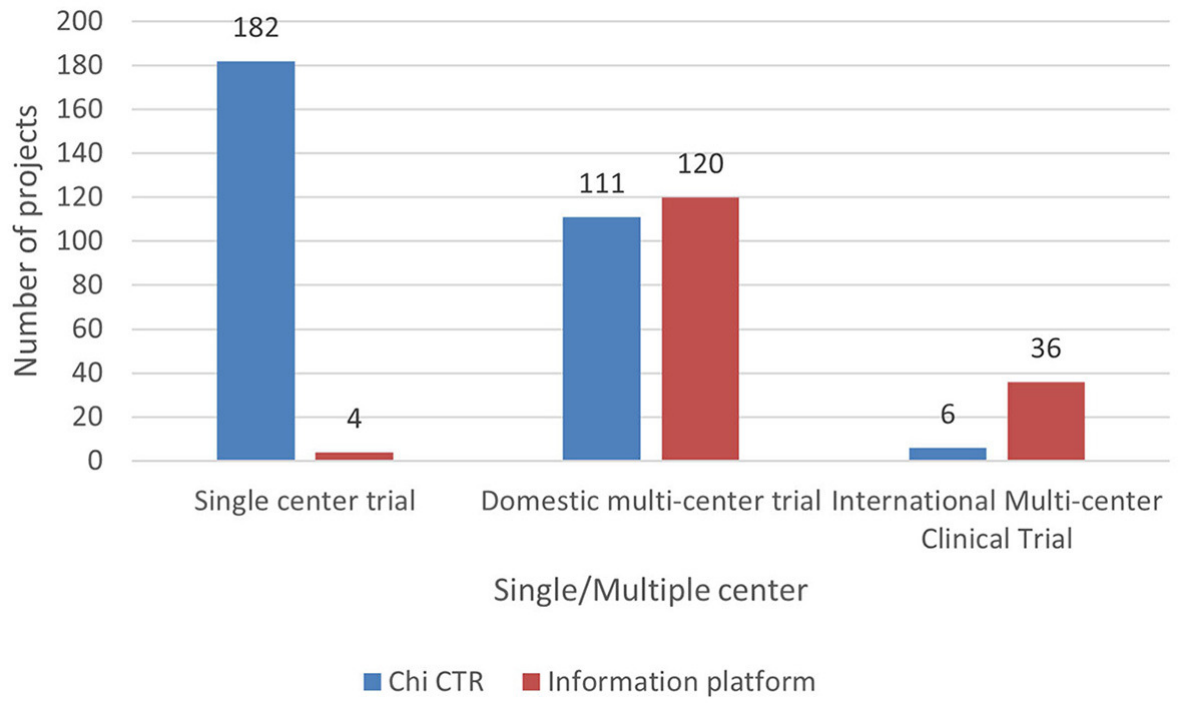
Distribution of pediatric drug clinical trials in children's hospitals in China.

Based on the scope of clinical trials, they are divided into single-center and multicenter trials. As some experimental projects include overseas research institutions, multicenter trials are divided into domestic and international multicenter trials. As can be observed from Figure 9, the clinical trial projects carried out by children's hospitals in China are mainly domestic multicenter projects, accounting for 50.33% (231/459) of the total projects. The clinical trials registered by the Chi CTR platform are mainly single-center trials. There is little cooperation with overseas research institutions. The first five clinical trial projects with a large number of participating research institutions (including domestic and international) are international multicenter trials, of which 247 are the most participating research institutions.

### 3.8 Ethics and research design

#### 3.8.1 Approval of the ethics committee

Of the 299 clinical trials included in the Chi CTR platform, 269 studies received ethics approval. Among the 160 clinical trials included in the Information Platform, 158 studies were approved by ethics, 53 studies were approved by ethics after revision, one study was approved by ethics after revision, and the pass rate of ethics approval was close to 66%. Two studies did not receive approval for ethics.

#### 3.8.2 Research design type

According to Chi CTR and Information Platforms' classification standard for clinical trial research design, the research design of pediatric drug clinical trials in children's hospitals in China is shown in Table 5. It is worth noting that two case-control studies, four cohort studies, and four cross-sectional studies are registered on the Chi CTR platform. Combined with specific information analysis, it is confirmed that they are experimental studies rather than observational studies, of which 6 are random parallel controls and 4 are non-random controls.

**Table 5:** Research and design of pediatric drug clinical trials in children's hospitals in China.

**Research design**	**Number**			**Total proportion**
	**Chi CTR**	**Information Platform**	**Total**	
Randomized parallel control	236	100	336	73.20
Single-arm	27	56	83	18.08
Non-randomized control	20	2	22	4.79
Sequential design	4	2	6	1.31
Factorial design	5	—	5	1.09
Randomized cross control	3	—	3	0.65
Semi-randomized control	3	—	3	0.65
Random sampling	1	—	1	0.22

#### 3.8.3 Blind design

The blindness of drug clinical trials included in this study is divided into single blindness, double blindness (including triple blindness), and openness (Table 6). There are 167 clinical trials on the Chi CTR platform that have not been blinded, but judging from the parallel control data in Table 5, they should all be double-blind designs.

**Table 6:** Blind design of pediatric drug clinical trials in children's hospitals in China.

**Blind design**	**Number**			**Total proportion**
	**Chi CTR**	**Information platform**	**Total**	
Unexplained	167	—	167	36.38
Double-blind and triple-blind	62 and 3	81	146	31.80
Open	58	74	132	28.76
Single-blind	9	4	14	3.05

## 4 Discussion

### 4.1 Rapid development of drug clinical trial research in children's hospitals in China despite current limitations

According to Weijuan and Ji's (16) analysis of clinical trial registration in the top 20 medical journals (CSCD) in China, only 7.40% of the prospective clinical studies published between 2017 and 2018 had a clinical trial registration number. This percentage significantly differs from that of prospective clinical studies published abroad during the same period, which stood at 67.40%. Yanjiao et al. (17) found that pediatric drug clinical trials accounted for merely 1.3% of all drug trials on the Information Platform from September 2013 to April 2022, falling far below the global level of 11.2%. Among these pediatric drug trials, children's hospitals organized or participated in approximately three-quarters (160 out of a total of 207). The overall strength of drug clinical trials and registration in Chinese children's hospitals is relatively weak based on data from both platforms; however, there has been an upward trend since 2018. As the number of clinical trials increases, so does the number of approved drugs available on the market. In line with this growth trend, according to a drug evaluation report in 2023, there was a significant increase in the approval granted for as many as 92 varieties specifically designed for children, compared to only 66 varieties approved in 2022.

The research findings demonstrate effective complementarity between the information obtained from both platforms: (A) primarily focusing on pre-marketing phase III clinical trials and post-marketing drug research stages conducted by children's hospitals, further confirming or enhancing drugs' known safety and effectiveness data; (B) while pilot projects on the Information Platform are mainly driven by enterprises or sponsors aiming for drug registration and listing goals, the Chi CTR platform pilot project is more centered around independent hospital-based or joint promotion-driven clinical research; and C. With the exception of four projects, the Information Platform primarily focuses on multicenter research (with overseas centers accounting for approximately one-third), while the Chi CTR platform predominantly conducts single-center research. Based on the data, Beijing Children's Hospital of Capital Medical University serves as an exemplary national or regional medical center-level children's hospital that plays a pivotal role in post-approval clinical and exploratory research. However, the overall participation of Chinese children's hospitals in drug clinical trials remains relatively low, necessitating continuous research to expedite the process of obtaining support from provincial regional children's hospitals for pediatric drug clinical trials. Simultaneously, it is imperative to enhance standardization and emphasize the significance of clinical trial registration within Chinese children's hospitals to establish a robust foundation for global information sharing under the purview of the World Health Organization.

### 4.2 Structural imbalances in some diseases/systemic pediatric disease, children's traditional Chinese medicine, and other clinical trials

According to the data, the diseases/systems involved in the drug clinical trials carried out by children's hospitals in China are mainly respiratory system, tumor, endocrine, and nutrition or metabolic diseases, and the clinical trials of the respiratory system still have a certain degree of aggregation. In recent years, the number of children with cancer in China has gradually increased and showed a trend toward younger age. Leukemia has become the most common childhood malignant tumor, accounting for approximately 32% of childhood cancers (18). Research on endocrine growth hormone deficiency has also received increasing attention. From the perspective of trial distribution structure, this is consistent with the actual dynamic situation of the pediatric disease spectrum, but there are shortcomings in clinical trial items involving the visual system. Given the current situation, the demand for drugs for myopia, growth promotion, and mental and behavioral disorders among children in China will increase rapidly in the future. Compared with general hospitals, children's hospitals in China have limited research on children's visual systems (accounting for 2.40%), thus we can consider paying more attention to the development of clinical trials of pediatric drugs involving visual systems.

Currently, chemical drugs are still dominant in drug clinical trials in China. Data show that children's hospitals in China mainly focus on chemical drug research on both the Chi CTR and Information Platform. Compared with traditional Chinese medicine (TCM), the number of research projects is the least, accounting for 13.07% (60/459); of which, Chi CTR accounts for 15.38% (46/299), and Information Platform accounts for 8.75% (14/160). In comparison, Chinese medicine also has exclusive advantages in pediatric respiratory, digestive, and other systems/diseases. Yang et al. (19) found that the information on the use of TCM for children in China is not complete, and the varieties of TCM for children that have been listed lack safety and effectiveness evaluation. It is recommended that children's hospitals leverage national policies to accelerate the clinical trials of TCM for children. This approach would enhance and effectively utilize the unique benefits of traditional Chinese medicine in protecting children's health.

### 4.3 Limited research on young children and age distribution differences in two platform projects

Data show that children, infants, and adolescents are the main subjects recruited in the clinical trials registered on the Chi CTR platform, while children and adolescents are the main subjects on the Information Platform.

Although there are few clinical drug trials involving newborns in children's hospitals, the Chi CTR platform lists approximately four times as many trials with full-term newborns (98 trials) as the Information Platform (25 trials). It can be seen that, while carrying out phase III and post-marketing drug research, children's hospitals have carried out more basic research on independent exploratory experiments/pre-experiments in the neonatal stage, where enterprises are less concerned about listing.

In terms of drug types, the number of clinical trials among all age groups showed more chemical drugs, followed by biological products and traditional Chinese medicine. This trend is especially notable in trials involving newborns and infants, which was similar to the overall pattern of drug clinical trials in China. The selection of drug types was directly related to factors such as children's diseases, dosage form suitability, and data extrapolation. In most cases, it cannot be directly extrapolated from older children to younger children, especially newborns need the support of pediatric clinical trial data of corresponding age groups (10). Therefore, regardless of the purpose of listing and registration or the need for clinical drug accessibility, we can consider a variety of funding sources or project channels to appropriately increase the clinical trials of special drugs for young children in children's hospitals.

### 4.4 Unbalanced geographical distribution and the radiation-driven efficiency of medical centers at national and regional levels

Data show that children's hospitals in Beijing, Shanghai, Chongqing, Zhejiang, and Jiangsu all rank among the top five in China in the number of drug clinical trials; of which, the number of clinical trials accounts for 52.07% of the total number of clinical trials. The top five children's hospitals with clinical trials are among the top 15 in the competitiveness ranking of children's hospitals in China (20). This reflects the objective geographical imbalance in the development of pediatric drug clinical trials in China, which is related to the distribution of children's hospitals, their medical resources, and the technical level. The driving and balanced attributes of the regional distribution of the National Children's Medical Center and the Pediatric Regional Medical Center need to be further highlighted. We can continue to strengthen the organization and leading attributes of the national and regional medical centers and optimize the balanced and driving attributes of the distribution of development factors, such as the coverage of multicenter experimental projects and the composition of ethics committees.

### 4.5 The research design of pediatric drug clinical trials fully linked with ethical risk control

The ethics committee mainly ensures that the rights and safety of the subjects are protected. From the data, we can see that there is a proportion of preliminary revisions in ethical review among the data from the two platforms. Children have complex physiological systems, and their physical and psychological characteristics vary at different ages (21). Problems such as difficulty recruiting subjects, weak cooperation in joining the group, high risk of falling off, and many unexpected reactions cause multiple challenges for the randomized parallel controlled trial (22). When conducting an ethical review of pediatric drug clinical trials in children's hospitals, we should not only pay attention to the risk factors that are not often considered in adult trials but also keep track of the review, increase the frequency of the review, and grasp the ethical risks (23) so as to protect the rights and interests of children and help to achieve the goals of pediatric drug clinical trials smoothly.

## 5 Conclusion

In summary, despite the overall increase in pediatric drug clinical trials conducted by children's hospitals in China, there are still several shortcomings; these include the adequacy of platform registration information, the scope of international multicenter trials, regionally balanced development, comprehensive coverage across disease/system and age groups, research on traditional Chinese medicine systems, basic research, and comprehensive trials for listed registered drug varieties. However, improvements are expected with the ongoing reform in the national evaluation and approval system, alongside incentives for pediatric drugs, strategies for the inheritance and innovation of traditional Chinese medicine, and favorable policies related to China's participation in the International Council for Harmonization of Technical Requirements for Pharmaceuticals for Human Use (ICH).

There are certain limitations in this study that need to be acknowledged. First, the registration information on the Chi CTR platform was provided independently by registrants, which may lead to inaccuracies and incompleteness, thus potentially compromising authenticity. However, starting in 2023, it is anticipated that there will be enhanced standardization, integrity, and timeliness of information available on this platform. Second, the Information Platform has comparatively less experimental data available, making it challenging to conduct comprehensive comparisons and analyses.

## Data availability statement

The datasets presented in this study can be found in online repositories. The names of the repository/repositories and accession number(s) can be found in the article/Supplementary material.

## Author contributions

YY: Conceptualization, Formal analysis, Methodology, Project administration, Visualization, Writing – original draft, Writing – review & editing. RJ: Data curation, Writing – original draft. GB: Conceptualization, Methodology, Writing – review & editing. QL: Conceptualization, Methodology, Resources, Writing – review & editing. YC: Formal analysis, Project administration, Resources, Writing – review & editing.
